# Cepharanthine synergistically promotes methylprednisolone pharmacodynamics against human peripheral blood mononuclear cells possibly via regulation of P-glycoprotein/glucocorticoid receptor translocation

**DOI:** 10.1186/s12906-024-04489-z

**Published:** 2024-05-11

**Authors:** Wencheng Xu, Shuhe Chen, Xiaoqin Wang, Jinwen Min, Sachiko Tanaka, Kenji Onda, Kentaro Sugiyama, Haruki Yamada, Toshihiko Hirano

**Affiliations:** 1 Department of Pharmacy, Hubei Provincial Hospital of Traditional Chinese Medicine, Wuhan, 430064 P. R. China; 2 Hubei Key Laboratory of Theory and Application Research of Liver and Kidney in Traditional Chinese Medicine, Affiliated Hospital of Hubei University of Chinese Medicine, Wuhan, P. R. China; 3 Hubei Province Academy of Traditional Chinese Medicine, Wuhan, P.R. China; 4 Department of Nephrology, Hubei Provincial Hospital of Traditional Chinese Medicine, Wuhan, P. R. China; 5 The First Clinical Medical College, Jinzhou Medical University, Jinzhou, P.R. China; 6 Department of Clinical Pharmacology, School of Pharmacy, Tokyo University of Pharmacy and Life Sciences, 1432-1 Horinouchi, Hachioji, Tokyo, 192-0392 Japan

**Keywords:** Cepharanthine, Glucocorticoid receptor, Methylprednisolone, P-glycoprotein, peripheral blood mononuclear cell

## Abstract

**Background:**

Cepharanthin^®^ alone or in combination with glucocorticoid (GC) has been used to treat chronic immune thrombocytopenia (ITP) since the 1990s. Cepharanthine (CEP) is one of the main active components of Cepharanthin^®^. The purpose of this study was to investigate the effects of CEP on GC pharmacodynamics on immune cells and analyse the possible action mechanism of their interactions.

**Methods:**

Peripheral blood mononuclear cells (PBMCs), T lymphocytic leukemia MOLT-4 cells and daunorubicin resistant MOLT-4 cells (MOLT-4/DNR) were used to evaluate the pharmacodynamics and molecular mechanisms. Drug pharmacodynamics was evaluated by WST-8 assay. P-glycoprotein function was examined by rhodamine 123 assay. CD4^+^CD25^+^Foxp3^+^ regulatory T cells and Th1/Th2/Th17 cytokines were detected by flow cytometry. P-glycoprotein expression and GC receptor translocation were examined by Western blot.

**Results:**

CEP synergistically increased methylprednisolone (MP) efficacy with the suppressive effect on the cell viability of PBMCs. 0.3 and 1 μM of CEP significantly inhibited P-glycoprotein efflux function of CD4^+^ cells, CD8^+^ cells, and lymphocytes (*P*<0.05). 0.03~3 μM of CEP also inhibited the P-glycoprotein efflux function in MOLT-4/DNR cells in a concentration-dependent manner (*P*<0.001). However, 0.03~3 μM of CEP did not influence P-glycoprotein expression. 0.03~0.3 μM of CEP significantly increased the GC receptor distribution from the cytoplasm to the nucleus in a concentration-dependent manner in MOLT-4/DNR cells. The combination did not influence the frequency of CD4^+^, CD4^+^CD25^+^ and CD4^+^CD25^+^Foxp3^+^ T cells or the secretion of Th1/Th2/Th17 cytokines from PBMCs. In contrast, CEP alone at 1 μM decreased the percentage of CD4^+^ T cell significantly (*P*<0.01). It also inhibited the secretion of IL-6, IL-10, IL-17, TNF-α, and IFN-γ.

**Conclusions:**

CEP synergistically promoted MP pharmacodynamics to decrease the cell viability of the mitogen-activated PBMCs, possibly via inhibiting P-glycoprotein function and potentiating GC receptor translocation. The present study provides new evidence of the therapeutic effect of Cepharanthin^®^ alone or in combination with GC for the management of chronic ITP.

**Supplementary Information:**

The online version contains supplementary material available at 10.1186/s12906-024-04489-z.

## Background

**Fig. 1:**
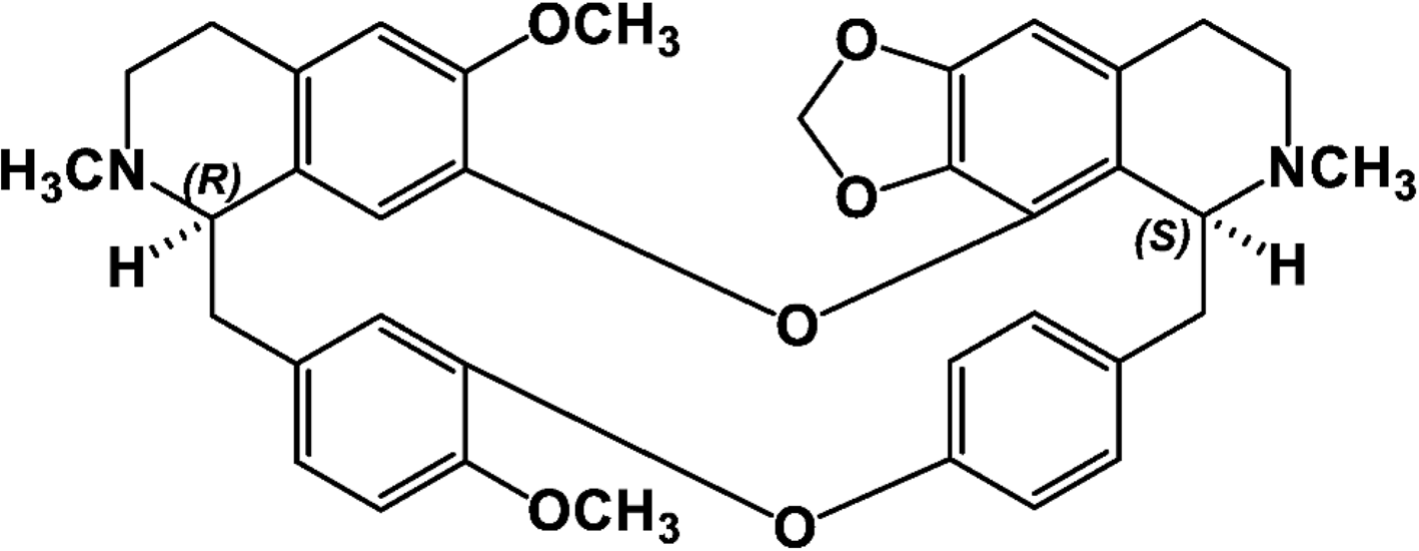
Chemical structure of cepharanthine (CEP)

Cepharanthin^®^ (Kaken Shoyaku Co. Ltd., Japan) is a complex of biscoclaurine alkaloids extracted from *Stephania cepharantha* HAYATA [1]. It has been approved for more than 70 years in Japan to treat a variety of acute and chronic diseases, such as venomous snakebites, radiation-induced leukopenia, alopecia ateata, and alopecia pityrodes [2, 3]. According to the description in the package insert, this drug mainly contains four kinds of natural compounds: cepharanthine (CEP, Fig. 1), isotetrandrine, berbamine, and cycleanine [4].

Immune thrombocytopenia (ITP) is an autoimmune bleeding disorder that is characterized by thrombocytopenia and normal or increased numbers of bone marrow megakaryocytes [5, 6]. It occurs in both adults and children, with a multimodal incidence with one peak in childhood, and second and third peaks in young adults and the elderly [7]. The 2011 American Society of Hematology guidelines recommend glucocorticoid (GC) therapy as the first-line treatment for chronic ITP [8, 9]. The side effect profile of GCs, including infection, weight gain, hypertension, and diabetes, has been an issue for some ITP patients, including pediatric patients [8, 10]. Several clinical observations in Japan have demonstrated that Cepharanthin^®^ combined with GC is useful in the management of chronic ITP [1, 8]. However, the underlying mechanism of the combination remains unclear.

P-glycoprotein is a transmembrane protein of 170 kD encoded by the multidrug resistance 1 (MDR-1) gene. It transports a variety of substrates, including GC, out of the immune cells [11]. Over-functional P-glycoprotein has been reported to play an important role in the pathogenesis of ITP and to reduce the immunosuppressive efficacy of GCs in non-responsive ITP patients [12]. Our previous study suggested that CEP strongly inhibited P-glycoprotein function in T lymphocytic leukemia daunorubicin resistant MOLT-4 cells (MOLT-4/DNR), whereas isotetrandrine and berbamine, the other two components of Cepharanthin^®^, showed extremely weak inhibitory effects on P-glycoprotein function [13].

Whether CEP influences GC pharmacodynamics *via* its interaction with P-glycoprotein in human immune cells remains unclear. Previous study indicates that T cells play a central role in ITP immunopathogenesis by initiating, propagating, and maintaining antiplatelet autoimmunity [5]. Concanavalin A (ConA) is recognized as a T-cell mitogen [14], thus, in the present study, a ConA-activated peripheral blood mononuclear cell (PBMC) culture procedure was used to evaluate the effects of CEP on GC pharmacodynamics. We also investigated the possible action mechanism of the combination, with a focus on P-glycoprotein/GC receptor (GR) translocation, aiming to provide new evidence of the therapeutic effect of Cepharanthin^®^ combined with GC for the management of chronic ITP.

## Methods

### Chemicals and reagents

CEP (HPLC purity: 99.1%, batch number: 0483686) was purchased from the Cayman Chemical Company. Methylprednisolone (MP) and verapamil (VP) were obtained from Sigma-Aldrich (St. Louis. Mo., USA). CEP, MP, and VP were dissolved in ethanol and stored at 4℃ until use. ConA was purchased from Seikagaku Kogyo Co. Tokyo, Japan. The WST-8 reagent was provided by Dojindo Molecular Technologies, Inc., Tokyo, Japan. The BD Cytometric Bead Array Human Th1/Th2/Th17 Cytokine Kit, FITC mouse anti-human CD4, APC mouse anti-human CD8 and CD25, and Alexa Fluor^®^ 488 mouse anti-human Foxp3 antibodies were obtained from BD Biosciences (San Jose, CA, USA). The anti-P-glycoprotein antibody was purchased from Kamiya Biomedical Company, Seattle, USA (1 μg/mL, # MC-208). Anti-GR antibody was obtained from Santa Cruz Biotechnology, INC, Texas, USA (G-5, dilution:1:1000, # sc-393232). The anti-β-actin antibody was provided by Proteintech Group, Rosemont, USA (1:5000, # 66009-1-lg). The anti-TATA binding protein (TBP) antibody was purchased from Abcam (Cambridge, UK; dilution:1:1000, # ab818). RPMI-1640 and fetal bovine serum (FBS) were obtained from Gibco BRL (Grand Island, NY, USA). All other reagents used were of the highest quality available from commercial vendors.

### Subjects

This study was approved by the Ethical Committee of Tokyo University of Pharmacy and Life Sciences, and written informed consent was obtained from all healthy subjects included in the study. The study included eight healthy subjects (four males and four females with a mean age of 34 years). These subjects had neither a history of immunological disorders nor a history of immunosuppressive drug use.

### Isolation of PBMCs and evaluation of drug effects in vitro

PBMCs were isolated from 20 mL of venous blood from healthy subjects as described previously [14–17]. In brief, heparinized blood was loaded into 4 mL of Ficoll-Hypaque (Nakarai Co., Japan) and centrifuged at 1300 × g for 20 min. PBMCs were collected, washed, and resuspended in RPMI 1640 medium containing 10% FBS, 1000,000 IU/L penicillin, and 100 mg/L streptomycin.

The density of the PBMC suspension was maintained at 1 × 10^6^ cells/mL. The cell suspension (196 μL) with or without the addition of 5 μg/mL ConA was seeded into the wells of a 96-well plate. Then, 2 μL of MP solution was added to obtain final MP concentrations of 0.1, 1, 10, and 100 ng/mL. Subsequently, 2 μL of the CEP solution was added to obtain final CEP concentrations of 0.03, 0.3, 1, and 3 μM. 4 μL of ethanol was added to the control wells. After incubation for 3 days in 5% CO_2_ at 37℃, 10 μL of WST-8 assay reagent solution was added to each well. The plates were further incubated for 3 hours. Optical density was measured at 450 nm (ref. 650 nm). The percentage of PBMC viability was calculated using the following formula: (Test‐Blank)/(Control‐Blank) × 100 (%). The IC_50_ values of MP were determined using GraphPad Prism 8 [16, 17]. The combination index was calculated using CompuSyn software, and a combination index < 1, 1, and > 1 represents synergistic, additive, and antagonistic effects, respectively [18].

### Human T lymphoblastoid leukemia cell culture

MOLT-4 cells, which express low levels of P-glycoprotein, were purchased from DS Pharma Biomedical Co., Ltd. (Osaka, Japan). MOLT-4/DNR cells, which are known to express large amounts of P-glycoprotein, were developed from the MOLT-4 cell line in our laboratory by exposing the parent cells to increasing concentrations of daunorubicin (DNR) over 3 months [18]. The expression of functional P-glycoprotein in these two cell lines was identified in our previous study [18]. MOLT-4 and MOLT-4/DNR cells were maintained in RPMI 1640 medium containing 10% FBS, 1000,000 IU/L penicillin, and 100 mg/L streptomycin [18].

### Evaluation of P-glycoprotein function

P-glycoprotein function in PBMCs, MOLT-4 cells, and MOLT-4/DNR cells was detected by rhodamine 123 (Rh123) efflux assay with flow cytometry, as described previously [16–18].

Briefly, PBMCs were incubated with 2 μM of Rh123 for 10 min in 5% CO_2_ at 37℃. After uptake of the dye, the cells were resuspended in Rh123‐free complete media with or without drugs. Subsequently, the cells were incubated for 180 min at 37°C to remove the dye out of cells. VP (5 μM) was used as the positive control. After the efflux period, PBMCs were incubated with FITC mouse anti-human CD4 and APC mouse anti-human CD8 antibodies. The cells were then resuspended in phosphate buffered saline (PBS) and kept on ice in the dark until analysis using FACS Canto TM II, as described previously (BD Biosciences, San Jose, CA, USA) [16, 17]. The percentage of Rh123 accumulation was determined to evaluate P-glycoprotein function of PBMCs.

After the cells were incubated with 2 μM of Rh123 in the presence or absence of drugs, Rh123 accumulation (%) during the dye-uptake period was determined to evaluate the P-glycoprotein function of MOLT-4 and MOLT-4/DNR cells. 1 mL of cell suspension containing 5 × 10^5^ cells was incubated with 2 μM Rh123 in the presence or absence of 0.03, 0.3, and 1 μM CEP for 1 h. After staining, the cells were washed and resuspended in ice-cold PBS. The intracellular Rh123 mean fluorescence intensity was examined using a FACS Canto TM II [18]. Data were analyzed using FlowJo software, version 10.4 [18].

### CD4^+^CD25^+^Foxp3^+^ regulatory T cell analysis

After PBMCs were cultured for 3 daysas described above, the culture supernatants were collected and stored at -80°C for measurement of cytokine concentrations (see below). PBMCs were stained with 10 μL FITC mouse anti-human CD4 and 10 μL APC mouse anti-human CD25 antibodies for 20 min at 37°C in the dark. After incubation, the cells were treated with human Foxp3 buffers A and B according to the manufacturer’s instructions. Next, 10 μL of Alexa Fluor® 488 mouse anti-human Foxp3 antibody was added, and the cell suspension was incubated for 30 min at 37°C in the dark. After washing the cells with PBS, 0.4 mL of staining buffer was added to the cell suspension, and then analyzed by flow cytometry. The data were analyzed with FACSCanto ™ II (BD Biosciences) using the BD FACSDiva™ software. CD4^+^ T cells in the lymphocyte fraction were gated, and the percentages of CD4^+^CD25^+^ and CD4^+^CD25^+^Foxp3^+^ cells in the CD4^+^ cell fraction were calculated as previously described [16].

### Evaluation of cytokines with cytometric bead array assay

The culture supernatant obtained as mentioned above was subjected to measure the concentrations of Th1/Th2/Th17 cytokines, IL-2, IL-4, IL-6, IL-10, IL-17, IFN-γ and TNF-α, by using Cytometric Bead Array assay, followed by flow cytometry, according to the manufacturer’s instructions [15, 16].

### Western blot analysis

Cytoplasmic and nuclear proteins were extracted using the Thermo Scientific NE‐PER Nuclear and Cytoplasmic Extraction Reagents (Pierce Biotechnology, Rockford, IL, USA). Whole cell protein was extracted using radioimmunoprecipitation assay (RIPA) buffer containing protease and phosphatase inhibitors (#A32961, Thermo Scientific). The protein concentration was quantified using the Pierce BCA Protein Assay Kit (#23227, Thermo Scientific). Western blotting was performed as described previously [17, 18]. 10 μg of whole cell or cytoplasmic protein lysate and 5 μg of nuclear protein lysate were separated using 10% sodium dodecyl sulfate-polyacrylamide gel electrophoresis and transferred onto polyvinylidene fluoride membranes respectively. The blots were cut prior to hybridisation with antibodies. The membrane signals were detected using a luminescent image analyzer (Fujifilm; LAS-3000; Fujifilm, Tokyo, Japan). Quantitative densitometry data were evaluated using ImageJ software (version 1.52e, National Institutes of Health, USA; http://imagej.nih.gov/ij) [17, 18].

### Statistical analysis

Differences between the values of drug-free controls and those obtained in the presence of serial concentrations of drugs were analyzed using Dunn's multiple comparison test. These analyses were performed using GraphPad PRISM 8.0 (GraphPad Software Inc., San Diego, CA, USA). In each case, a two-sided *P* value <0.05 was considered significant.

## Results

### Effects of CEP on GC pharmacodynamics in mitogen-activated PBMCs

**Fig. 2:**
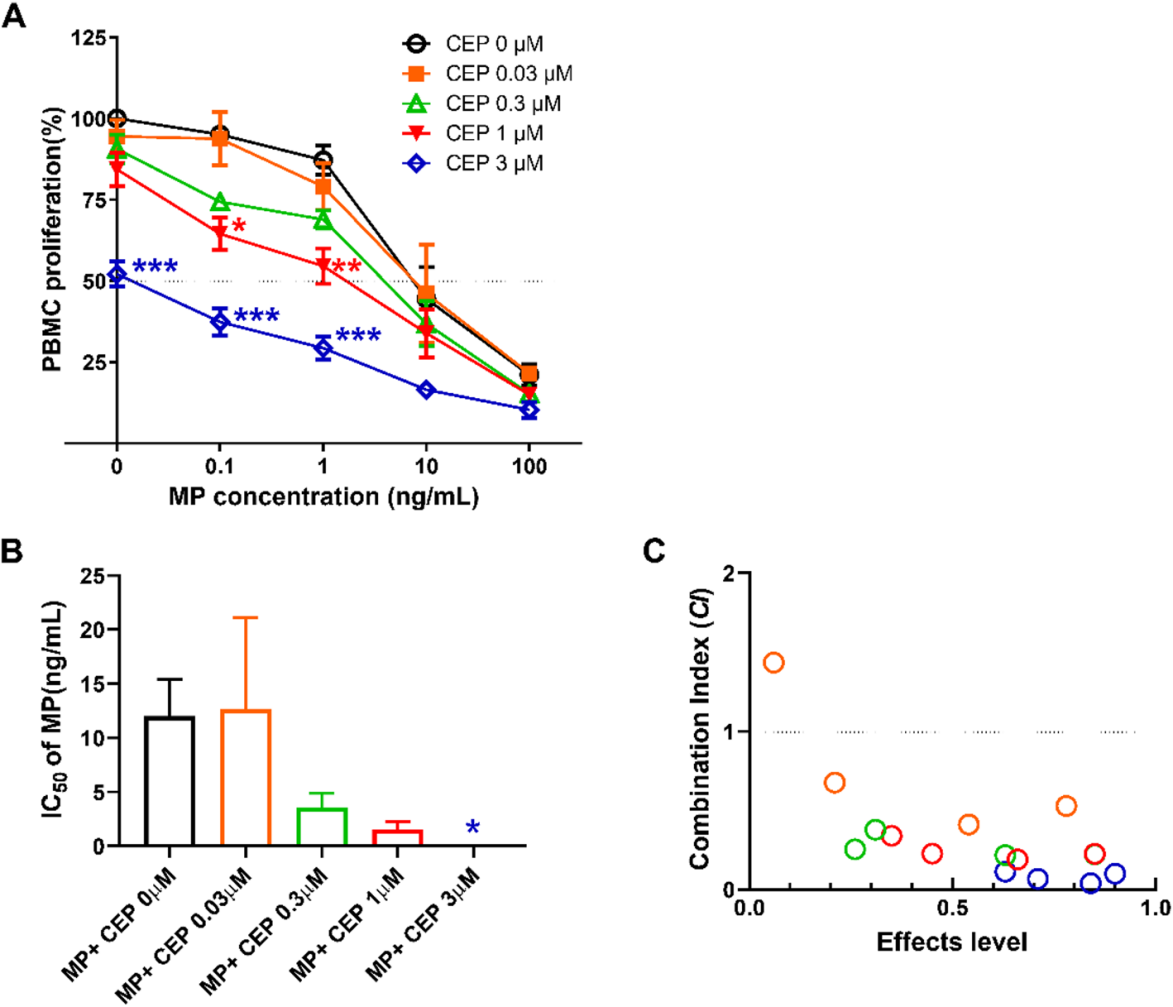
Effects of CEP on the pharmacodynamics of MP. **A** Suppressive effect of the combination of MP and CEP on the cell viability of PBMCs. **B** IC_50_ values of MP in the presence or absence of CEP. **C** Combination index of MP and CEP

As shown in Fig. 2A, 0.03~3 μM of CEP significantly potentiated MP pharmacodynamics to decrease the cell viability of ConA-activated PBMCs (*P*<0.05) in a concentration-dependent manner. Treatment with 3 μM CEP alone significantly decreased the cell viability of ConA-activated PBMCs (*P*<0.001). The IC_50_ value of MP alone was 12.01 ± 3.40 ng/mL. The combination of CEP at 0.03 μM with MP had little synergistic effect. However, the IC_50_ values of MP, combined with 0.3, 1, and 3 μM of CEP, were 3.58 ± 1.32, 1.53 ± 0.70, and >0.1 ng/mL, respectively, which was lower than that of MP alone (CEP 0 μM) (Fig. 2B). CEP decreased the IC_50_ values of MP in a concentration-dependent manner, and the additional effect of 3 μM CEP was statistically significant (Fig. 2B). According to the combination indices shown in Fig. 2C, the effects of MP combined with CEP were synergistic, as most of the combination indices were lower than 1.

### Effects of CEP on the P-glycoprotein function in PBMCs

**Fig. 3:**
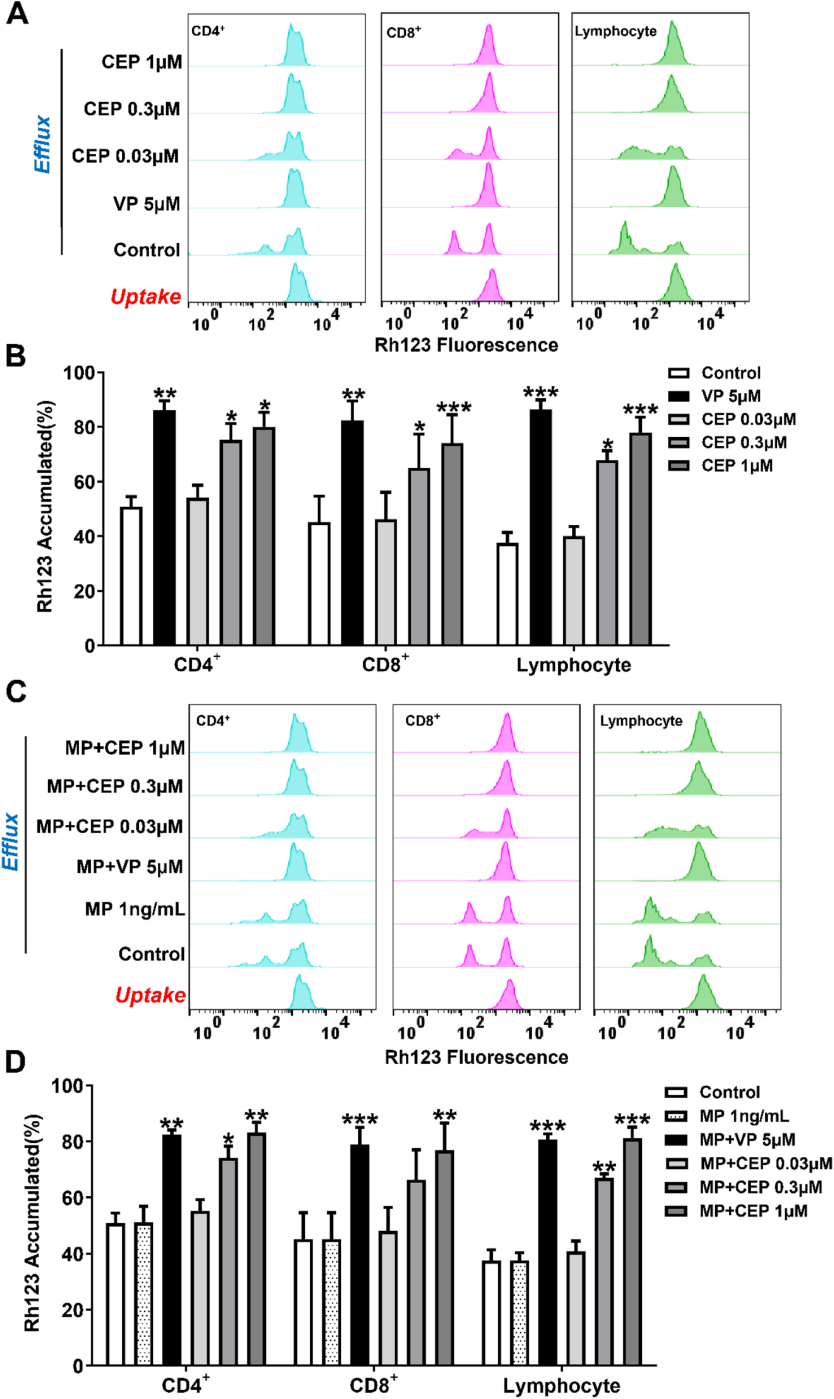
Effects of CEP on Rh123 accumulation in human PBMCs. **A**, **B** The data of PBMCs treated by CEP alone. **C**, **D** The data of PBMCs treated by MP combined with CEP

As shown in Fig. 3A, the fluorescence intensities of Rh123 in CD4^+^cells, CD8^+^ T cells, and lymphocytes were detected as signal peaks and remained in the right area (high-intensity level) in the uptake group. After efflux, the fluorescence intensities of these signal peaks decreased, and two signal peaks of Rh123 were generated in the control groups because P-glycoprotein excludes Rh123 out of cells. When PBMCs were treated with 5 μM VP, a P-glycoprotein inhibitor, the fluorescence intensity of Rh123 was significantly maintained in each cell subset (*P*<0.01, Fig. 3A and B). 0.03 μM CEP seemed to have little influence on the efflux function of P-glycoprotein in PBMCs. However, 0.3 and 1 μM CEP significantly inhibited the P-glycoprotein efflux function and maintained Rh123 fluorescence (*P*<0.05, Fig. 3A and B). On the other hand, 1 ng/mL MP did not affect Rh123 accumulation in each cell subset, suggesting that 1 ng/mL MP did not inhibit P-glycoprotein function (Fig. 3C and D). 1 ng/mL of MP also did not affect the inhibitory efficacies of CEP and VP on P-glycoprotein efflux function, since the fluorescence intensities of Rh123 did not change when PBMCs were treated by MP combined with CEP or VP (Fig. 3C and D).

### Effects of CEP on the P-glycoprotein function and expression in MOLT-4 and MOLT-4/DNR cells

**Fig. 4:**
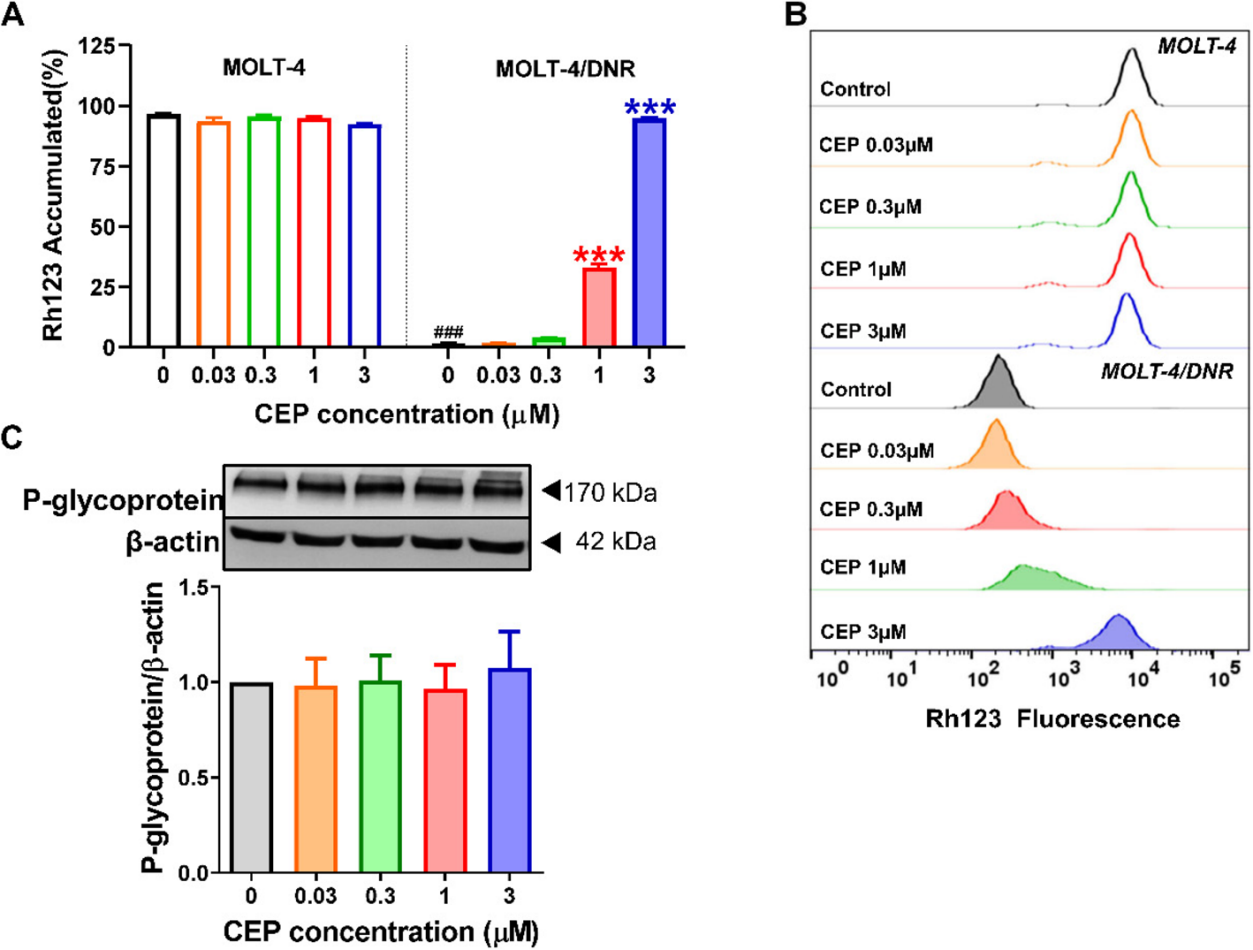
Effects of CEP on Rh123 accumulation and P-glycoprotein expression in MOLT-4 and MOLT-4/DNR cells. **A**, **B** The data of Rh123 accumulation. **C** P-glycoprotein expression in MOLT-4/DNR cells treated by CEP

MOLT-4 cells express little amount of P-glycoprotein [18], therefore, CEP, even at 3 μM, did not influence Rh123 accumulation in these cells (Fig. 4A and B). In contrast, MOLT-4/DNR cells expressed large amounts of P-glycoprotein [18], and Rh123 accumulation was significantly decreased due to P-glycoprotein function (*P*<0.001, Figs. 4A and B). However, CEP increased Rh123 accumulation in MOLT-4/DNR cells in a concentration-dependent manner (*P*<0.001, Fig. 4A). Accordingly, the fluorescence signal of Rh123 shifted to the right side in a concentration-dependent manner (Fig. 4B). Whereas, 0.03~3 μM of CEP did not influence the expression of P-glycoprotein in MOLT-4/DNR cells after treatment for 2 days (Fig. 4C).

### Effects of CEP on the GR translocation in MOLT-4/DNR cells

**Fig. 5:**
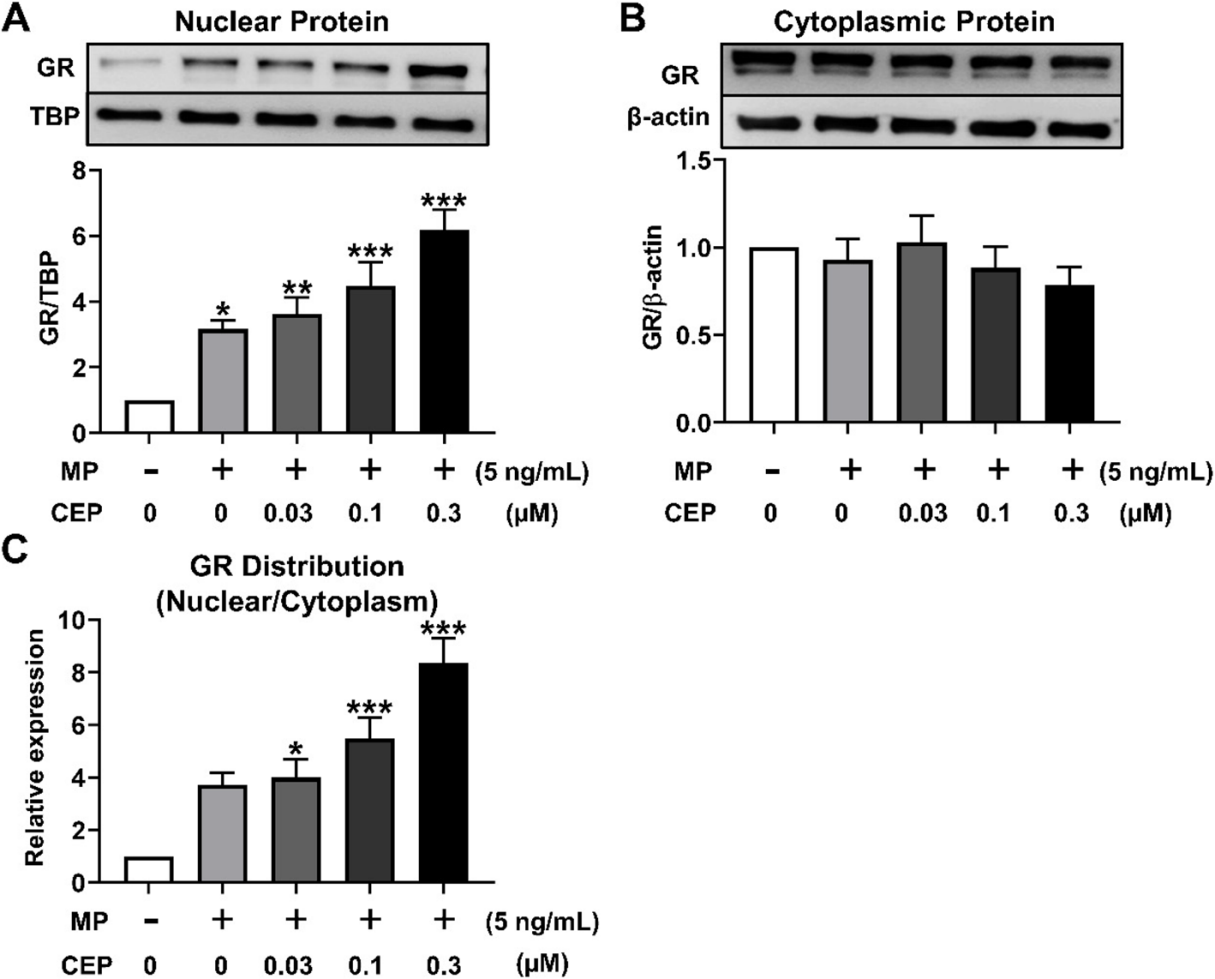
Effects of CEP on GR translocation in MOLT-4/DNR cells. **A** GR expression in the nuclear extracts of cells. **B** GR expression in the cytoplasmic extracts of cells. **C** GR distribution in nuclear and cytoplasm

The translocation of GR into the nucleus is recognized as an important process in GC pharmacodynamics [17]. As shown in Fig. 5A, 5ng/mL MP significantly stimulated nuclear GR expression in MOLT-4/DNR cells (*P*<0.05). 0.03~0.3 μM CEP potentiated GR translocation into the nucleus in MOLT-4/DNR cells in a concentration-dependent manner (*P*<0.01) (Fig. 5A). Accordingly, the cytoplasmic expression of GR decreased after treatment with MP combined with CEP in a concentration-dependent manner (Fig. 5B). Finally, 0.03~0.3 μM of CEP significantly increased the GR distribution from the cytoplasm to the nucleus in a concentration-dependent manner (*P*<0.05) (Fig. 5C).

### Effects of combination of CEP and MP on the frequencies of CD4^+^, CD4^+^CD25^+^ and CD4^+^CD25^+^Foxp3^+^ cells

**Fig. 6:**
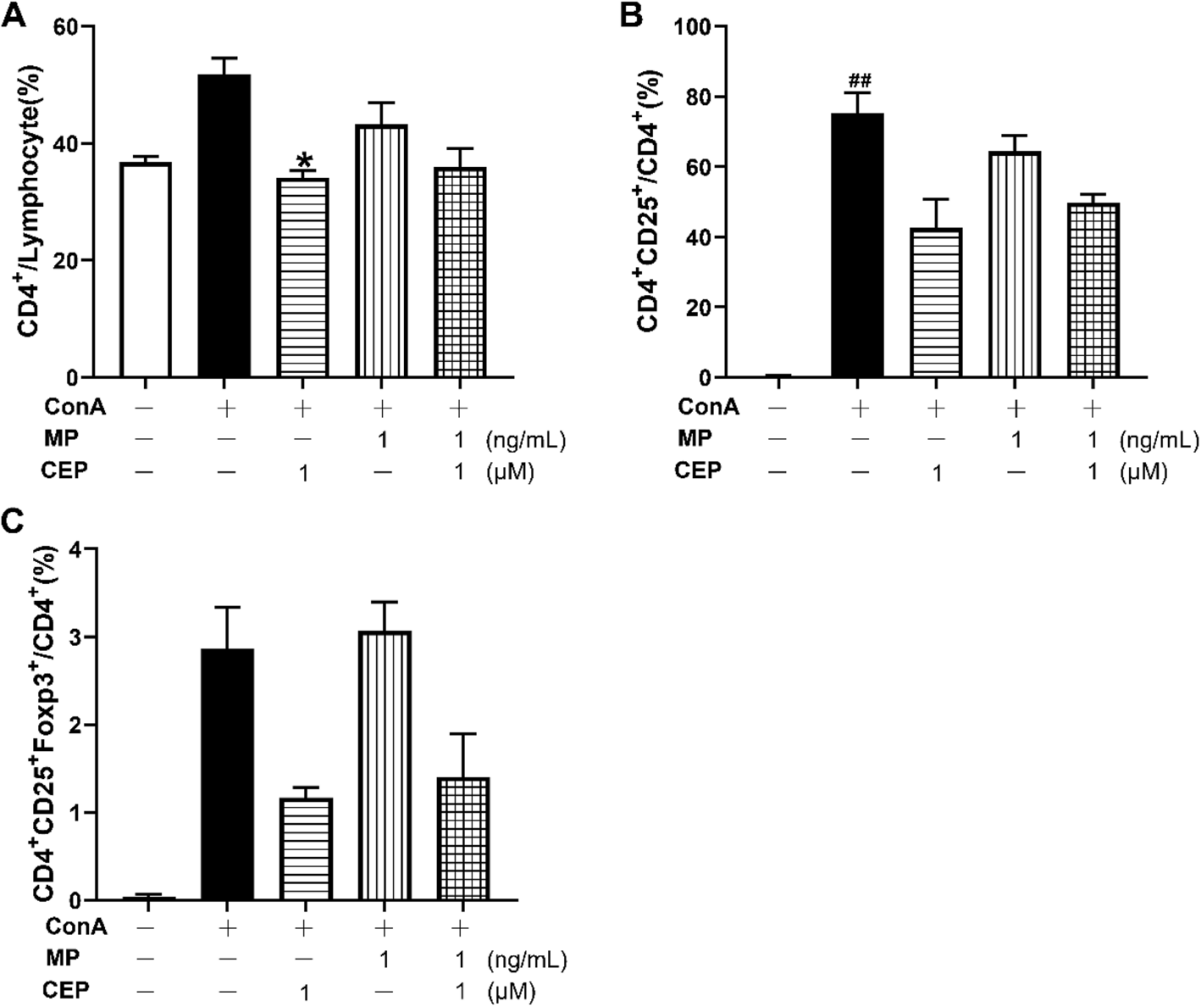
Effects of CEP and MP on the frequencies of CD4^+^, CD4^+^CD25^+^ and CD4^+^CD25^+^Foxp3^+^ cells. **A** Percentage of CD4^+^ in lymphocyte. **B** Percentage of CD4^+^ CD25^+^ in CD4^+^. **C** Percentage of CD4^+^ CD25^+^Foxp3^+^ in CD4^+^

As shown in Fig. 6A, 1μM CEP alone significantly decreased the percentage of CD4^+^ T cells in lymphocytes (*P*<0.05). However, no synergistic effects of CEP (1 μM) or MP (1 ng/mL) were observed. ConA significantly increased the percentage of CD4^+^CD25^+^cells in CD4^+^ cells (*P*<0.01). Treatment with 1 μM CEP alone suppressed the frequency of CD4^+^CD25^+^ cells in CD4^+^ cells (Fig. 6B). However, the combination of CEP (1 μM) and MP (1 ng/mL) did not show a synergistic inhibitory effect on the frequency of CD4^+^CD25^+^CD4^+^ cells (Fig. 6B). Meanwhile, no significant influence on the frequency of CD4^+^CD25^+^Foxp3^+^ cells in CD4^+^ cells was observed after PBMCs were treated with 1 μM CEP, 1 ng/mL MP, or their combination (Fig. 6C).

### Effects of combination of CEP and MP on cytokine production in mitogen-activated PBMCs

**Fig. 7:**
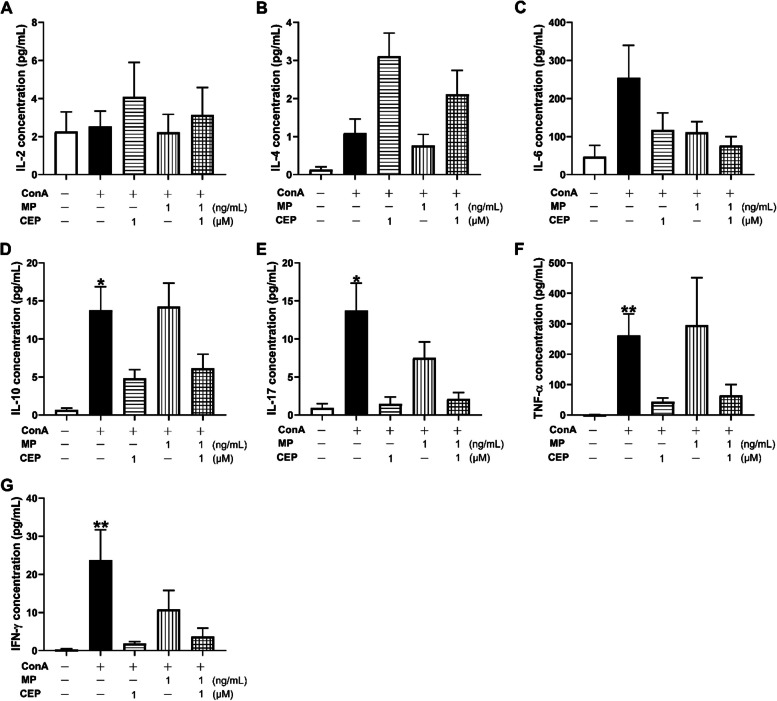
Effects of CEP and MP on the secretion of Th1/Th2/Th17 cytokines. **A** IL-2, (**B**) IL-4, (**C**) IL-6, (**D**) IL-10, (**E**) IL-17, (**F**) TNF-α and (**G**) IFN-γ concentrations in the supernatant of PBMC culture system

ConA significantly stimulated the secretion of IL-10, IL-17, TNF-α, and IFN-γ in PBMCs (*P*<0.05, Fig. 7D-G). 1 μM of CEP tended to inhibits the secretion of these cytokines. However, no synergistic effects on cytokine secretion were observed when PBMCs were treated with 1 μM CEP combined with 1 ng/mL MP. Both 1 μM CEP and 1 ng/mL MP showed similar suppressive effects on the secretion of IL-6, and the combination tended to show stronger inhibitory efficacy, although the effects were not statistically significant (Fig. 7C). The effects of these drugs on IL-2 and IL-4 secretion were weak (Fig. 7A-B).

## Discussion

Although Cepharanthin^®^ has been used to treat ITP since the 1990s and clinical trials revealed that Cepharanthin^®^ alone or combined with GC was useful for the management of pediatric chronic ITPs [3, 8, 19], the underlying mechanism of the combination remains unclear. Data from the present study firstly demonstrated that CEP synergistically increases MP pharmacodynamics to decrease the cell viability of ConA-activated PBMCs, possibly via the regulation of P-glycoprotein/GR translocation.

In the present study, we observed that 0.03~3 μM of CEP synergistically decreased the IC_50_ values of MP by inhibiting the cell viability of mitogen-activated PBMCs in a concentration-dependent manner (Fig. 2). Meanwhile, 0.03~1 μM of CEP were observed to inhibit the P-glycoprotein function of CD4^+^cells, CD8^+^ cells, and lymphocytes significantly in concentration dependent manners (Fig. 3). However, 1 ng/mL MP did not show any influence on P-glycoprotein function of these immune cells, which was consistent with the observations of our previous study [16]. Accordingly, it is possible to consider that CEP increases the intracellular concentration of MP in PBMCs by inhibiting P-glycoprotein efflux function.

Next, we examined the modulatory effect of CEP on GR nuclear translocation. Our previous study revealed that MP induces GR translocation in both leukemic Jurkat T cells and normal human PBMCs suggesting that T-lymphocytic leukemia cells are suitable to mimic ConA-activated PBMCs to study GR signalling pathway [17]. To evaluate the influence of P-glycoprotein on GR translocation in T cells, MOLT-4 cells with less amount of P-glycoprotein and the sub cell line MOLT-4/DNR with a large amount of P-glycoprotein were investigated and P-glycoprotein was confirmed to influence the GR translocation in T cells [18]. Thus, we used T-lymphocytic leukemia MOLT-4 cells and MOLT-4/DNR cells to evaluate the regulatory activity of CEP on P-glycoprotein/GR translocation in the present study. As shown in Fig. 4, CEP significantly inhibited the P-glycoprotein function of MOLT-4/DNR cells, which was consistent with the results obtained from PBMCs (Fig. 3**).** However, CEP at 1 μM showed a stronger suppressive effect on the P-glycoprotein function of PBMCs than MOLT-4/DNR cells (Figs. 3A and 4A). These results might indicate that the amount of P-glycoprotein expressed in MOLT-4/DNR cells was much higher than that in PBMCs. In contrast, CEP at 0.03~3 μM did not influence P-glycoprotein expression, suggesting that CEP mainly regulates P-glycoprotein function rather than protein expression. CEP potentiated GR nuclear translocation (Fig. 5), and therefore, it could be postulated that CEP inhibits P-glycoprotein function, increases the intracellular concentration of MP, and enhances GR translocation from the cytoplasm into the nucleus.

We also analyzed the effects of CEP and MP on the frequency of CD4^+^, CD4^+^CD25^+^ and CD4^+^CD25^+^Foxp3^+^ T cells. CD4^+^CD25^+^ cells are recognized as a marker on the surface of activated T lymphocytes [20]. As shown in Fig. 6B, ConA significantly stimulated the expression of CD25 (*P*<0.01), suggesting that the PBMCs were activated. Therefore, it was reasonable to conclude that ConA significantly stimulated the secretion of IL-10, IL-17, TNF-α, and IFN-γ (*P*<0.05, Fig. 7D-G). Although 1 μM of CEP combined with 1 ng/mL of MP significantly inhibited the proliferation of PBMCs (Fig. 2A), this combination did not significantly block the activation of T cells in PBMCs (Fig. 6B). This information indicates that inhibition of the expression of CD25, which is known as the IL-2 receptor, is not the main mechanism underlying the synergistic effects of the combination of MP and CEP. ConA activated PBMCs to increase the relative number of CD4^+^ T cells in lymphocytes, and 1 μM of CEP suppressed this activation (Fig. 6A). This finding may explain the therapeutic efficacy of Cepharanthin^®^ for the management of pediatric chronic ITPs, as CD4^+^ T cell proliferative responses were observed in cultures of PBMCs obtained from ITP patients in contrast to healthy donors [8, 21]. On the other hand, 1 μM of CEP tended to inhibit the secretion of IL-6, IL-10, IL-17, TNF-α, and IFN-γ from activated PBMCs (Fig. 7C-G), which would help to relieve theTh1/Th2/Th17 immune responses, since these cytokines were reported to contribute to the pathogenesis of ITP [21]. However, neither significant nor synergistic results against CD4^+^ T cell activation were observed after treatment with 1 μM of CEP combined with 1 ng/mL of MP (Fig. 6). Accordingly, we did not observe synergistic inhibitory effects of the combination on the secretion of Th1/Th2/Th17 cytokines (Fig. 7). CD4^+^CD25^+^Foxp3^+^ T cells have been reported to inhibit lymphocyte proliferation of lymphocytes [22]. However, in the present study, the combination did not increase the percentage of CD4^+^CD25^+^Foxp3^+^ T cells (Fig. 6C).

CEP, a natural bisbenzylisoquinoline alkaloid, has been used in the clinic for more than 70 years [23]. It has a variety of medicinal properties, including antioxidant, anti-inflammatory, immunomodulatory, antitumoral, and antiviral effects [23, 24]. Recently, CEP and its analogues were reported to show broad-spectrum anti-coronavirus activities [25]. According to our best knowledge, it was the novel finding that CEP synergistically potentiated the anti-proliferative function of GC possibly via the regulation of P-glycoprotein/GR translocation. Similar activities were observed on tetrandrine as we reported before [16, 18]. Meanwhile, both CEP and tetrandrine induced apoptosis through caspase cascade regulation, cell cycle arrest, MAPK activation and PI3K/Akt/mTOR signal modification in GC resistant human leukemia Jurkat T cells [26, 27]. However, the present study demonstrated that CEP selectively inhibited the efflux function of P-glycoprotein with little influence on the protein expression, which is totally different with tetrandrine [18]. These information hints that structure-activity relationship of bisbenzylisoquinoline alkaloids potential of increasing GC sensitivity via regulating P-glycoprotein deserves further study.

A limitation of our study is the lack of PBMCs to confirm the synergistical effect of CEP and MP and their action mechanism. It is possible to further investigate the role of CEP on MP pharmacodynamics and their action mechanism by using PBMCs with over-functional P-glycoprotein isolated from non-responsive ITP patients [12]. Although, Cepharanthin^®^ combined with GC were confirmed to be useful in the management of chronic ITP [1, 8], we look forward to new clinical reports which compare the effects of CEP and Cepharanthin^®^ combined with GC for non-responsive ITP patients.

## Conclusions

In summary, CEP synergistically increased MP pharmacodynamics to suppress T cell mitogen-activated PBMCs, possibly via regulation of P-glycoprotein/GR translocation. However, the combination did not influence the frequency of CD4^+^, CD4^+^CD25^+^ and CD4^+^CD25^+^Foxp3^+^ T cells in PBMCs or secretion of Th1/Th2/Th17 cytokines. CEP at 1 μM significantly decreased the percentage of CD4^+^ T cells and it also tended to inhibit the secretion of IL-6, IL-10, IL-17, TNF-α, and IFN-γ. The present study provides new evidence of the therapeutic efficacy of Cepharanthin^®^ alone or in combination with GC for the management of chronic ITP.

### Supplementary Information

Supplementary Material 1. 

## Data Availability

The data used during the current study are available from the corresponding author on reasonable request.

## Abbreviations

CEP: Cepharanthine
ConA: Concanavalin A
DNR: Daunorubicin
FBS: Fetal bovine serum
Fig: Figure
GC: Glucocorticoid
GR: Glucocorticoid receptor
ITP: Immune thrombocytopenia
MP: Methylprednisolone
PBMC: Peripheral blood mononuclear cell
PBS: Phosphate buffered saline
Rh123: Rhodamine 123
VP: Verapamil
