# Supplementary material for: Cepharanthine synergistically promotes methylprednisolone pharmacodynamics against human peripheral blood mononuclear cells possibly via regulation of P-glycoprotein/glucocorticoid receptor translocation
Source: BMC Complement Med Ther. 2024 May 11;24:186. doi: 10.1186/s12906-024-04489-z (PMC11088782; doi:10.1186/s12906-024-04489-z)
Supplement: Supplementary file 1 — Supplementary Material 1. [file 12906_2024_4489_MOESM1_ESM.pptx]

## Slide 1
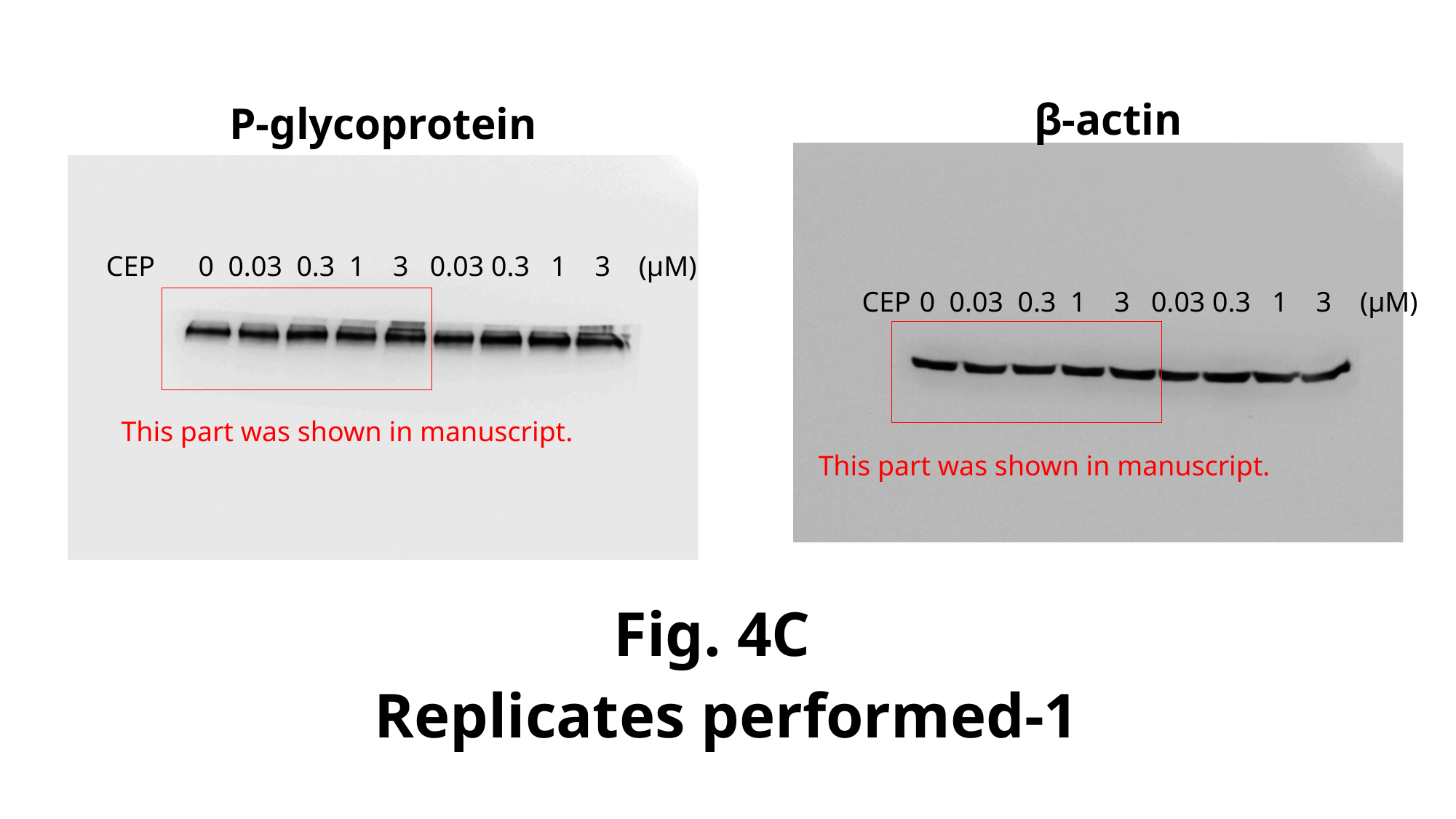

β-actin
P-glycoprotein
CEP
 0 0.03 0.3 1 3 0.03 0.3 1 3 (μM)
This part was shown in manuscript.
CEP
 0 0.03 0.3 1 3 0.03 0.3 1 3 (μM)
This part was shown in manuscript.
Fig. 4C
Replicates performed-1

## Slide 2
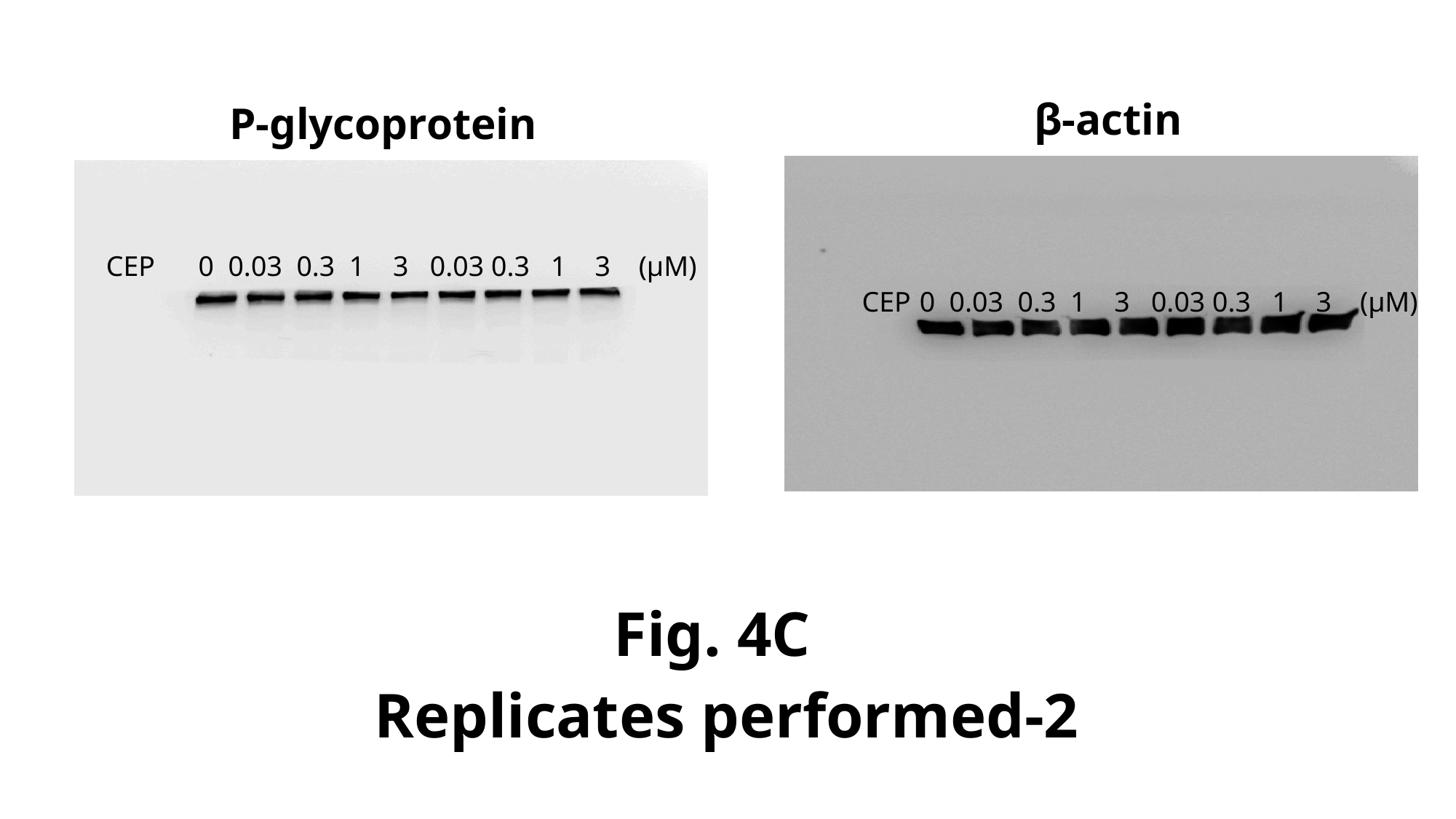

β-actin
P-glycoprotein
CEP
 0 0.03 0.3 1 3 0.03 0.3 1 3 (μM)
CEP
 0 0.03 0.3 1 3 0.03 0.3 1 3 (μM)
Fig. 4C
Replicates performed-2

## Slide 3
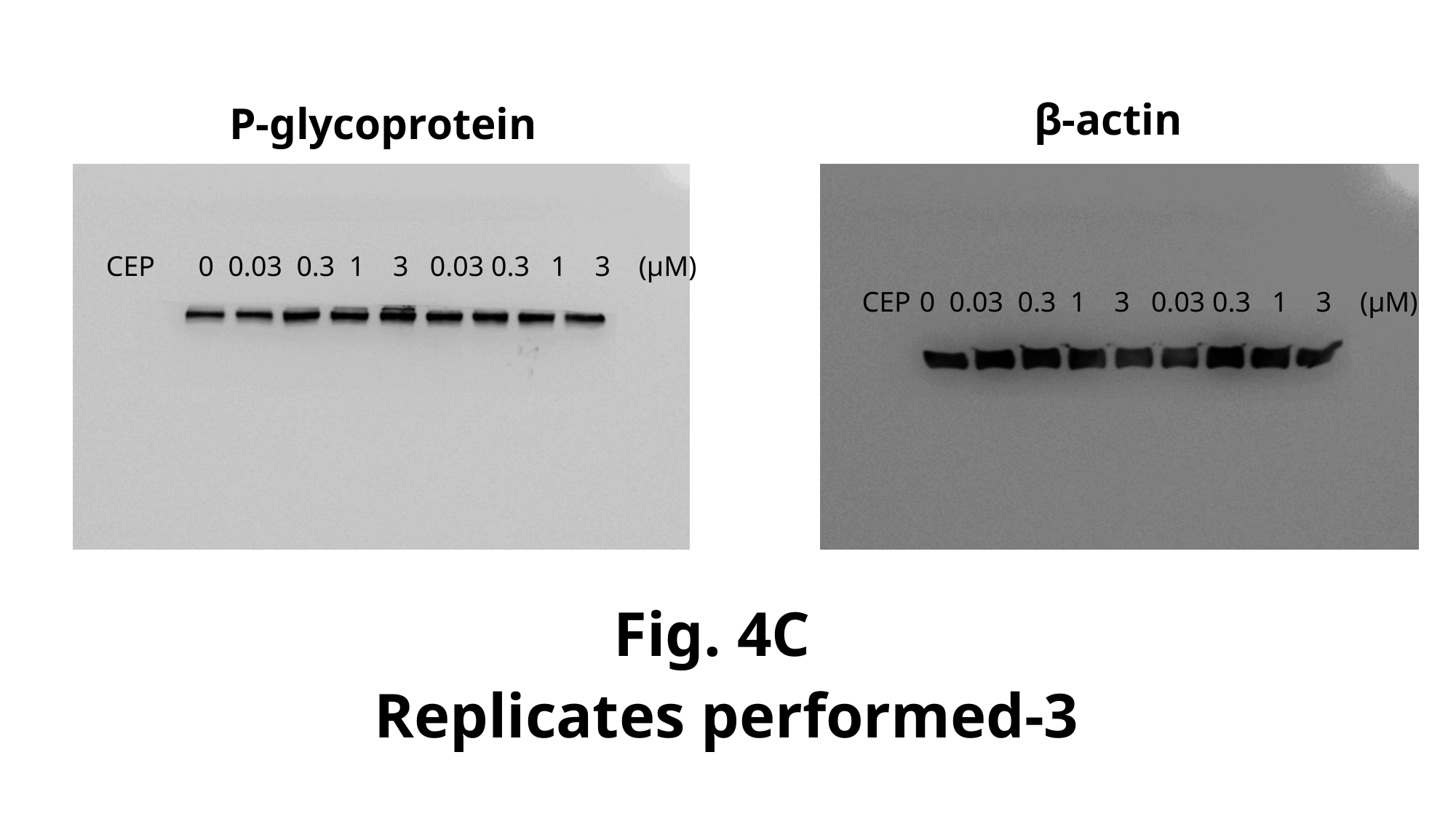

β-actin
P-glycoprotein
CEP
 0 0.03 0.3 1 3 0.03 0.3 1 3 (μM)
CEP
 0 0.03 0.3 1 3 0.03 0.3 1 3 (μM)
Fig. 4C
Replicates performed-3

## Slide 4
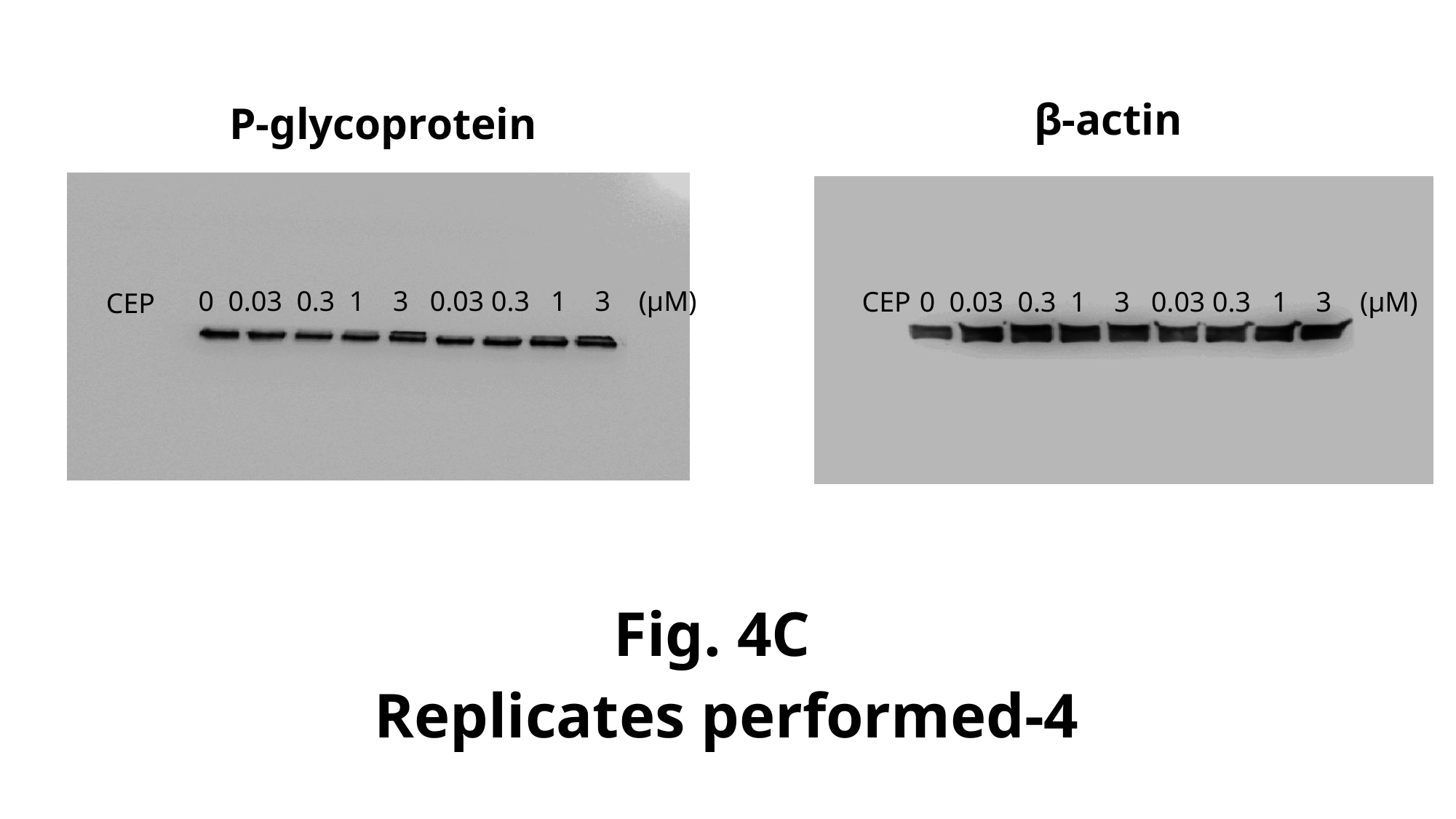

β-actin
P-glycoprotein
 0 0.03 0.3 1 3 0.03 0.3 1 3 (μM)
CEP
 0 0.03 0.3 1 3 0.03 0.3 1 3 (μM)
CEP
Fig. 4C
Replicates performed-4

## Slide 5
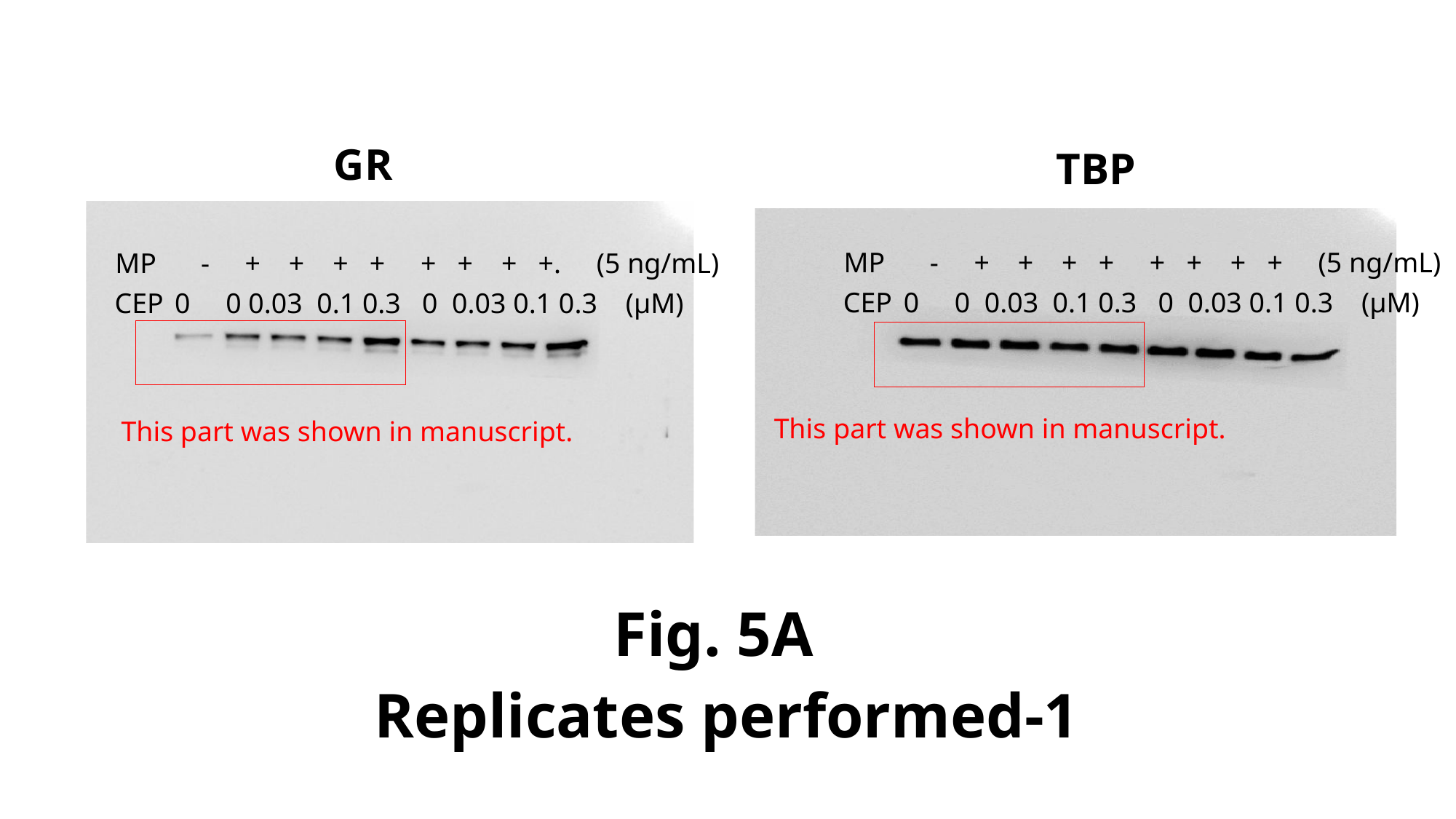

GR
TBP
MP
 - + + + + + + + + (5 ng/mL)
MP
 - + + + + + + + +. (5 ng/mL)
CEP
 0 0 0.03 0.1 0.3 0 0.03 0.1 0.3 (μM)
CEP
 0 0 0.03 0.1 0.3 0 0.03 0.1 0.3 (μM)
This part was shown in manuscript.
This part was shown in manuscript.
Fig. 5A
Replicates performed-1

## Slide 6
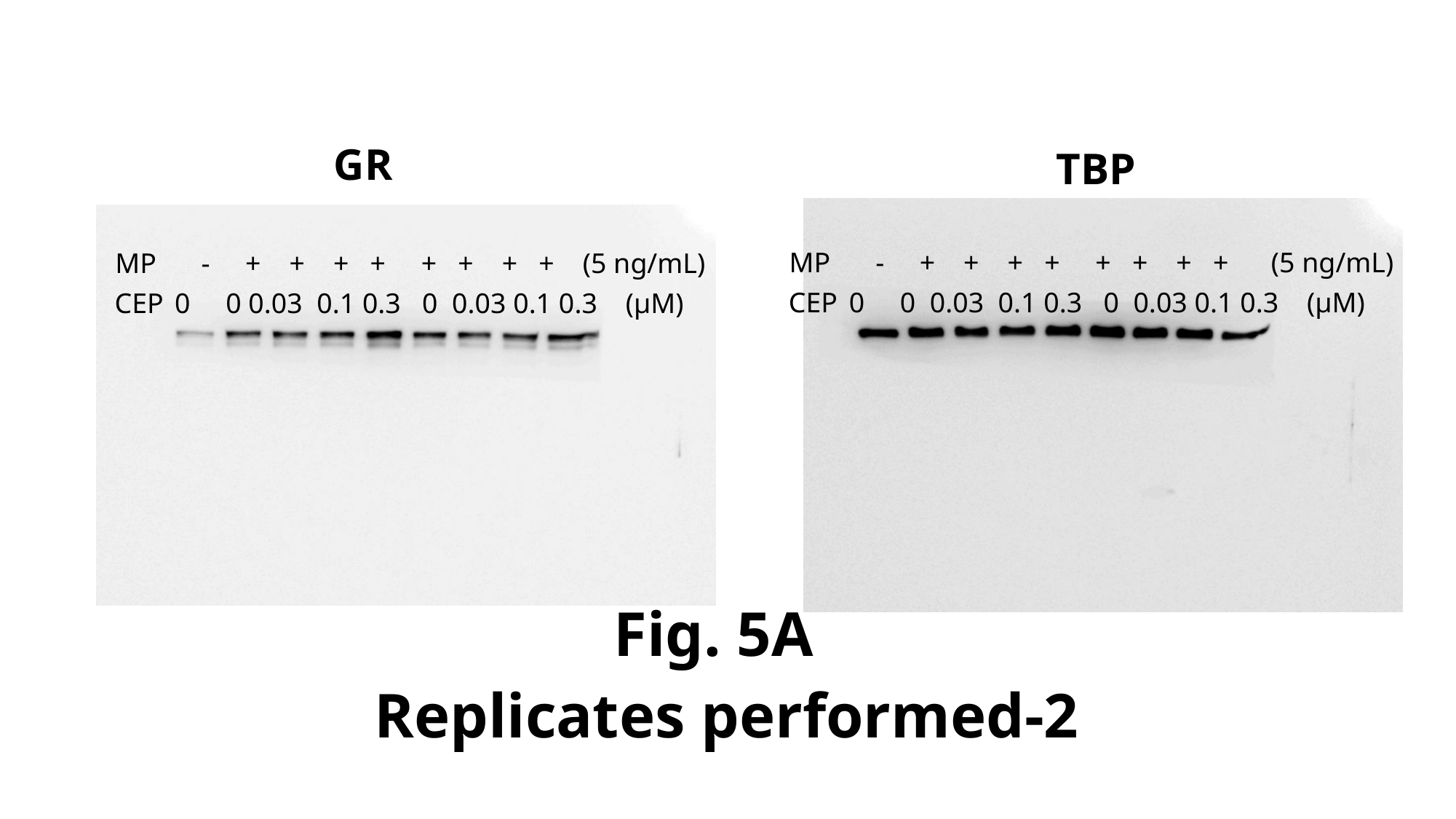

GR
TBP
MP
 - + + + + + + + + (5 ng/mL)
MP
 - + + + + + + + + (5 ng/mL)
CEP
 0 0 0.03 0.1 0.3 0 0.03 0.1 0.3 (μM)
CEP
 0 0 0.03 0.1 0.3 0 0.03 0.1 0.3 (μM)
Fig. 5A
Replicates performed-2

## Slide 7
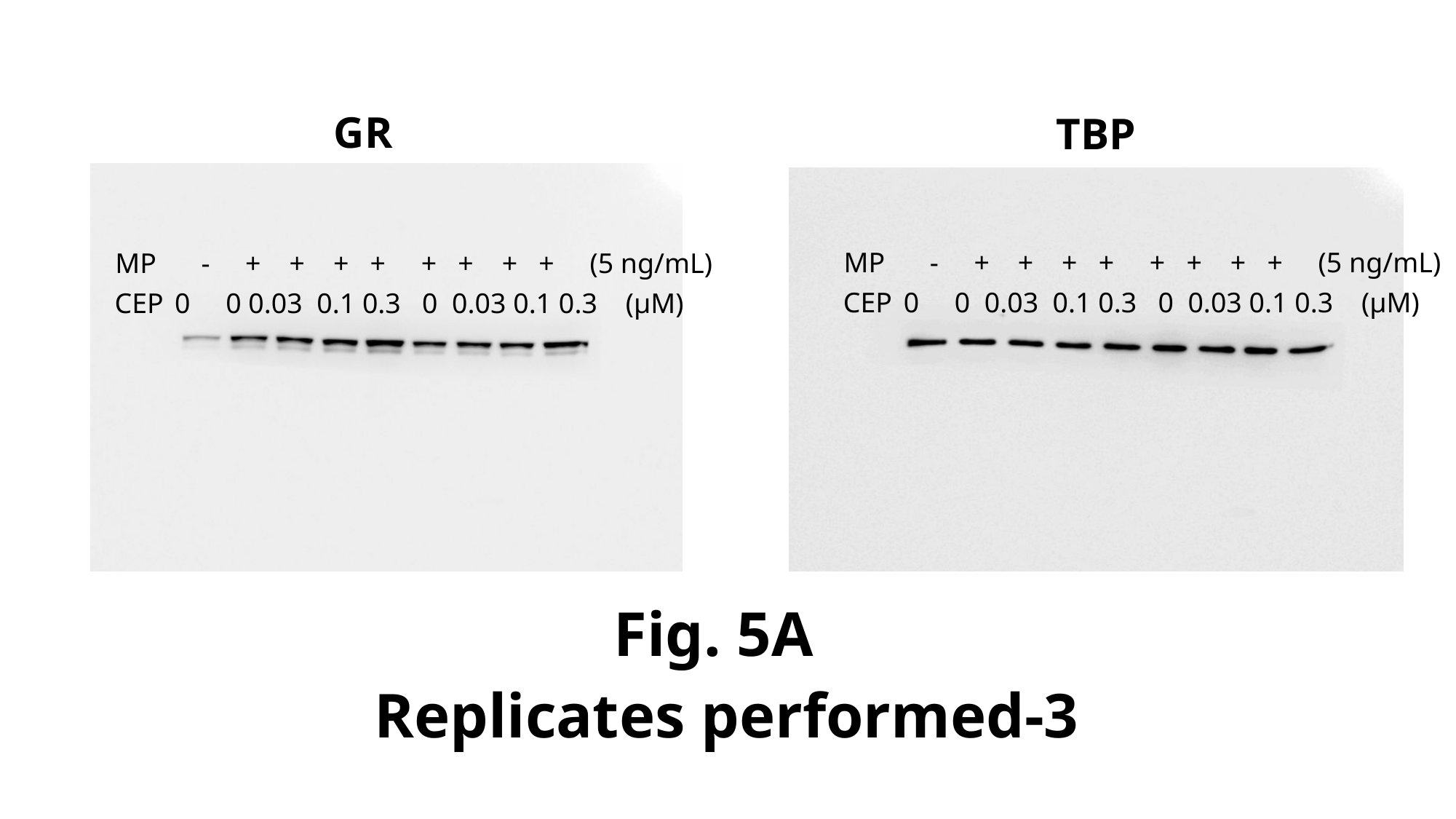

GR
TBP
MP
 - + + + + + + + + (5 ng/mL)
MP
 - + + + + + + + + (5 ng/mL)
CEP
 0 0 0.03 0.1 0.3 0 0.03 0.1 0.3 (μM)
CEP
 0 0 0.03 0.1 0.3 0 0.03 0.1 0.3 (μM)
Fig. 5A
Replicates performed-3

## Slide 8
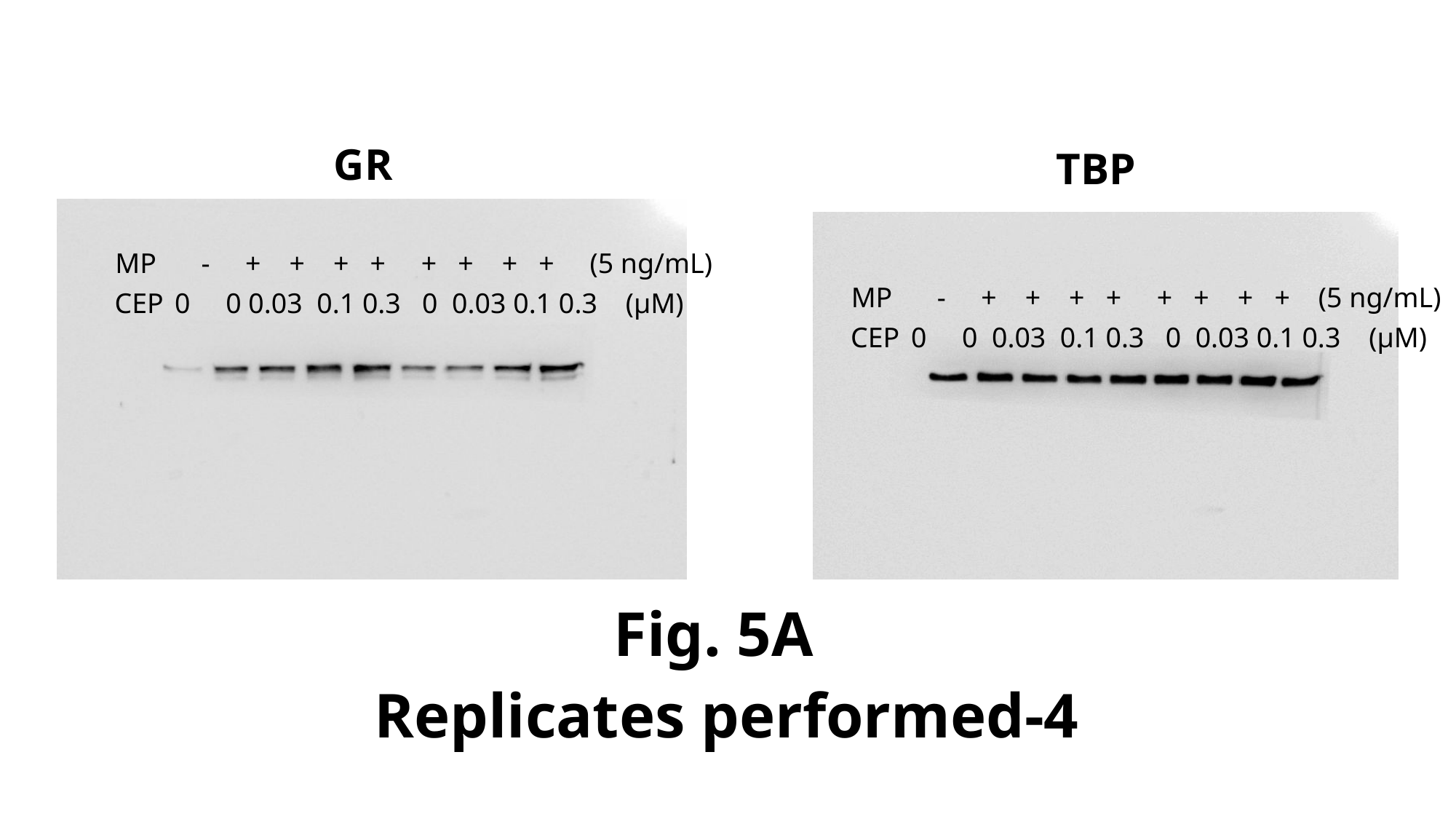

GR
TBP
MP
 - + + + + + + + + (5 ng/mL)
MP
 - + + + + + + + + (5 ng/mL)
CEP
 0 0 0.03 0.1 0.3 0 0.03 0.1 0.3 (μM)
CEP
 0 0 0.03 0.1 0.3 0 0.03 0.1 0.3 (μM)
Fig. 5A
Replicates performed-4

## Slide 9
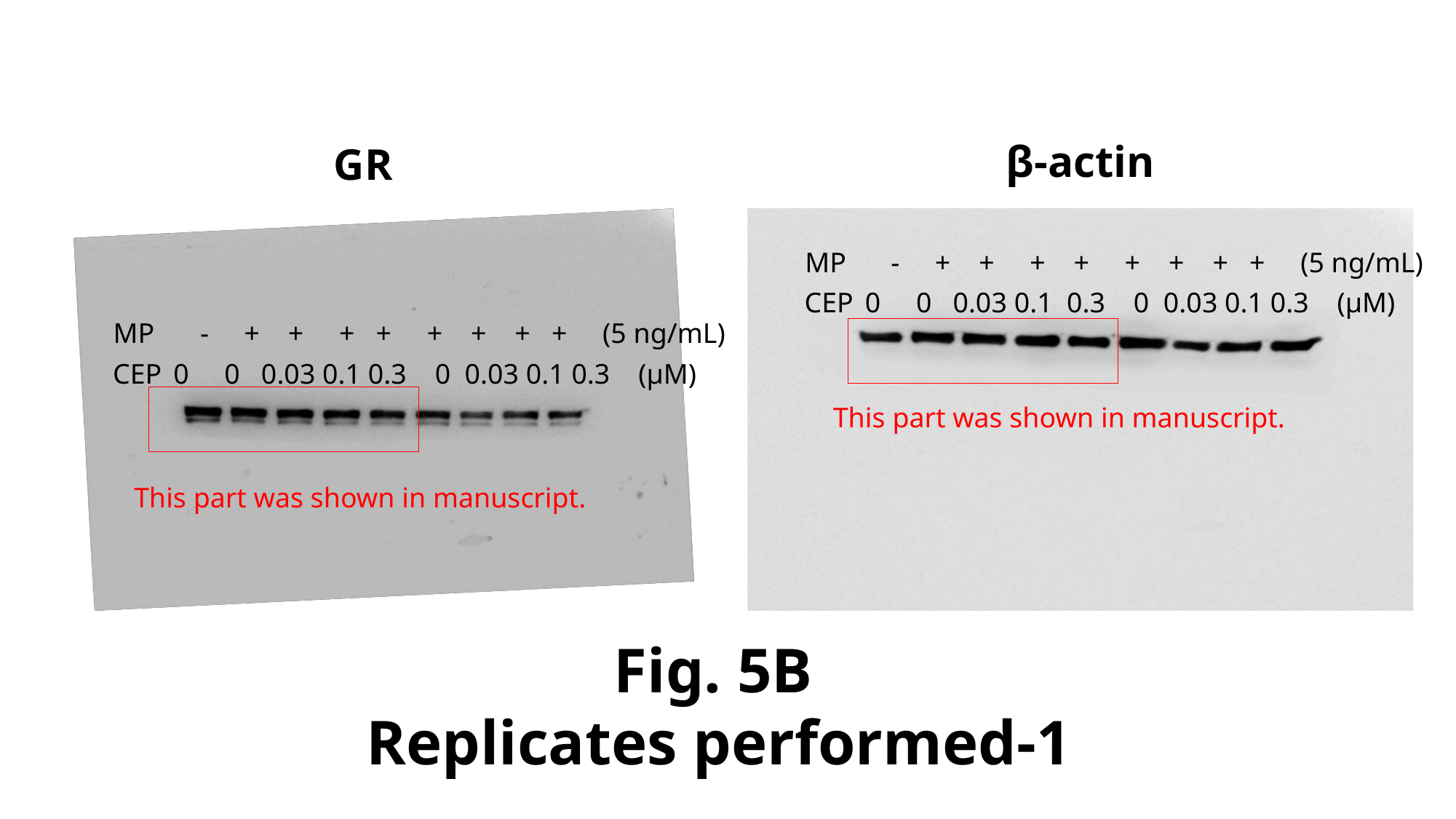

β-actin
GR
MP
 - + + + + + + + + (5 ng/mL)
CEP
 0 0 0.03 0.1 0.3 0 0.03 0.1 0.3 (μM)
MP
 - + + + + + + + + (5 ng/mL)
CEP
 0 0 0.03 0.1 0.3 0 0.03 0.1 0.3 (μM)
This part was shown in manuscript.
This part was shown in manuscript.
Fig. 5B
Replicates performed-1

## Slide 10
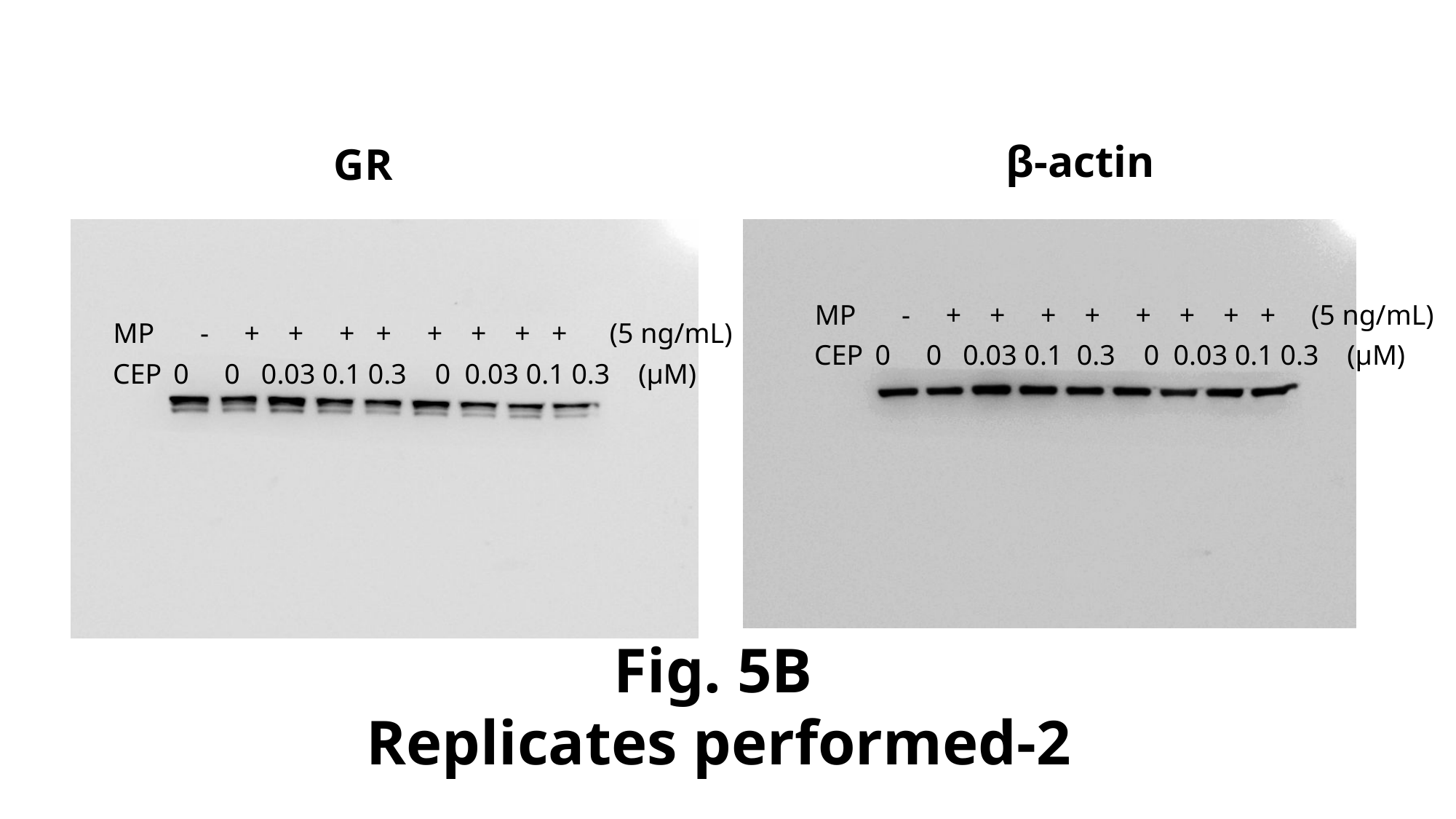

β-actin
GR
MP
 - + + + + + + + + (5 ng/mL)
MP
 - + + + + + + + + (5 ng/mL)
CEP
 0 0 0.03 0.1 0.3 0 0.03 0.1 0.3 (μM)
CEP
 0 0 0.03 0.1 0.3 0 0.03 0.1 0.3 (μM)
Fig. 5B
Replicates performed-2

## Slide 11
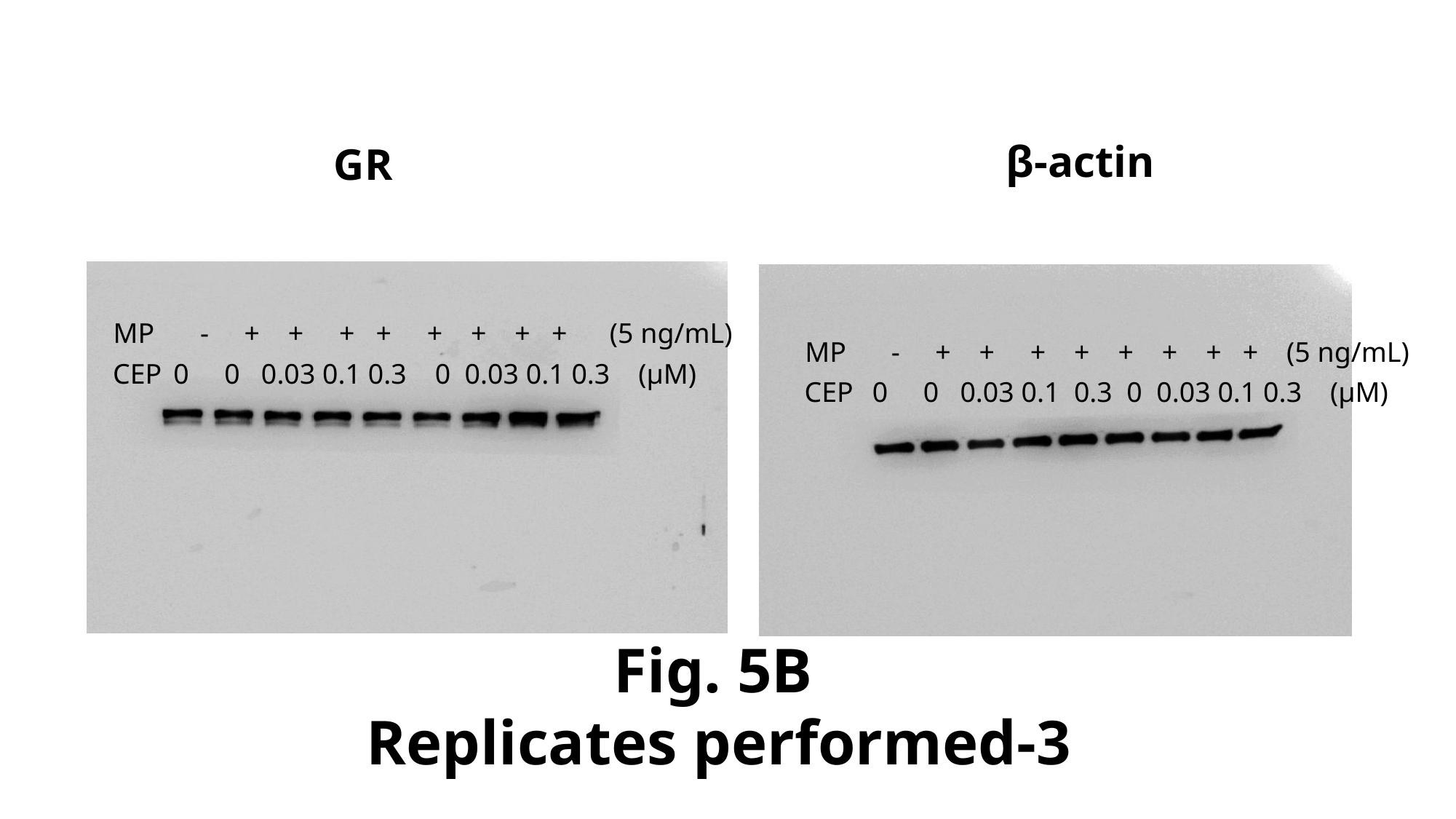

β-actin
GR
MP
 - + + + + + + + + (5 ng/mL)
MP
 - + + + + + + + + (5 ng/mL)
CEP
 0 0 0.03 0.1 0.3 0 0.03 0.1 0.3 (μM)
CEP
 0 0 0.03 0.1 0.3 0 0.03 0.1 0.3 (μM)
Fig. 5B
Replicates performed-3

## Slide 12
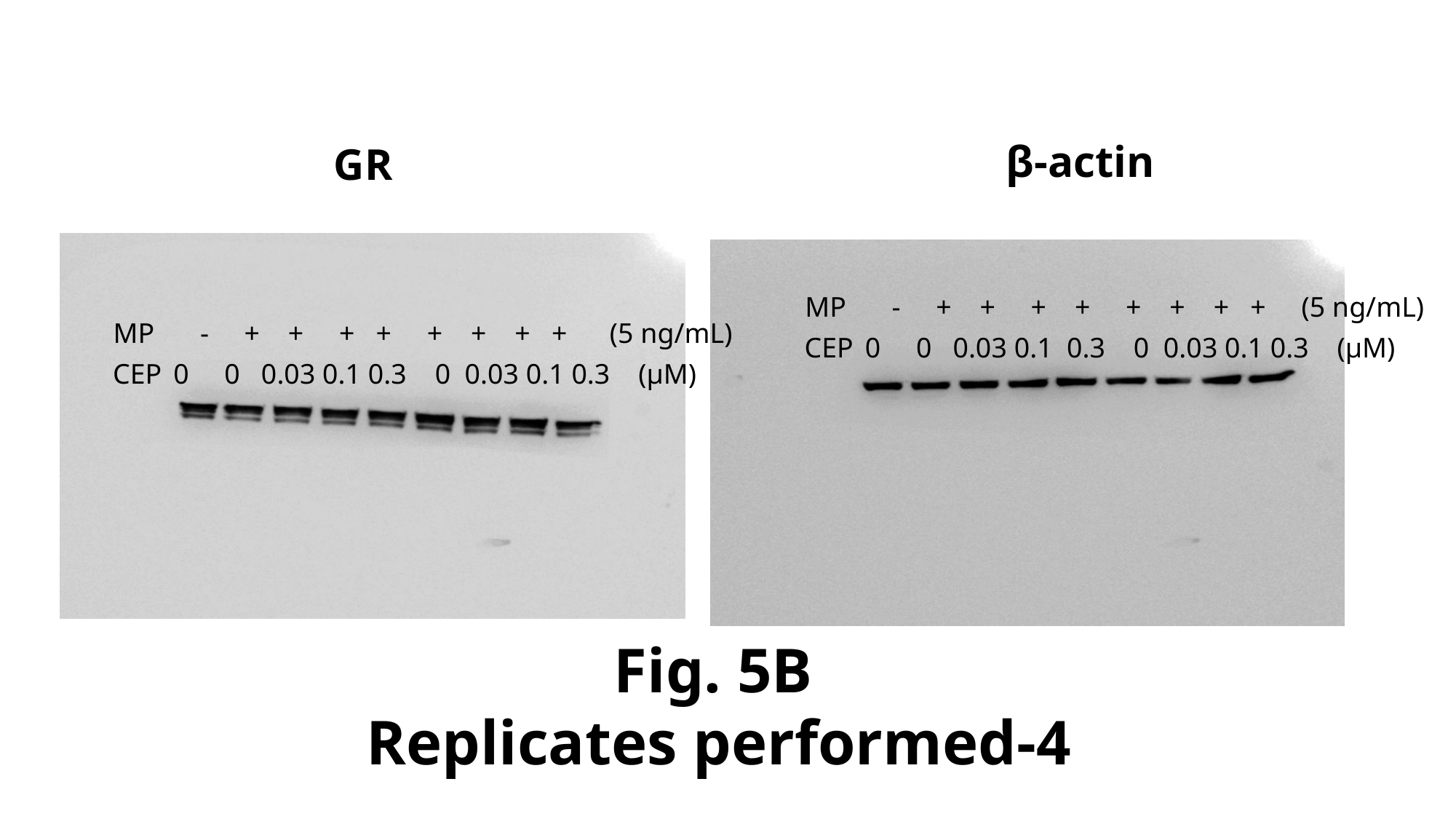

β-actin
GR
MP
 - + + + + + + + + (5 ng/mL)
MP
 - + + + + + + + + (5 ng/mL)
CEP
 0 0 0.03 0.1 0.3 0 0.03 0.1 0.3 (μM)
CEP
 0 0 0.03 0.1 0.3 0 0.03 0.1 0.3 (μM)
Fig. 5B
Replicates performed-4
